# Distal penile pyogenic granuloma: A case report

**DOI:** 10.1002/ccr3.8659

**Published:** 2024-03-08

**Authors:** Gideon Safiel Mwasakyalo, Frank Bright, Orgenes Jasper Mbwambo, Bartholomeo Nicholaus Ngowi, Daniel Mwakibibi, Jasper Saidi Mbwambo, Musa Raymond Majura, Evans Azina Sanga, Alex Mremi

**Affiliations:** ^1^ Department of Urology Kilimanjaro Christian Medical Centre Moshi Tanzania; ^2^ Faculty of Medicine Kilimanjaro Christian Medical University College Moshi Tanzania; ^3^ Department of Anaesthesia and Critical care Ministry of Health Social Services (hMOHSS) District Hospital Keetmanshoop Kharas Namibia; ^4^ Department of Pathology Kilimanjaro Christian Medical Centre Moshi Tanzania

**Keywords:** botryomycosis hominis, granuloma pyogenicum, lobular capillary hemangioma, surgical excision, telangiectasis granuloma

## Abstract

**Key Clinical Message:**

The mainstays of treatment for granuloma pyogenicum include careful evaluation of any penile growth, thorough excision of the polypoid, histological examination, and close monitoring to check relapse and management.

**Abstract:**

Pyogenic granuloma is an acquired noncancerous vascular proliferation that arises from the mucosa and skin, seldom subcutaneously or intravascularly. It is also referred to as telangiectasis granuloma or lobular capillary haemangioma. The risk factors include vascular abnormalities, medicines, hormones, and microtrauma. We discussed the case of a 24‐year‐old man who had a poorly managed ventral distal penile polypoid lesion at a peripheral hospital. Upon further histopathological examination, the diagnosis of pyogenic granuloma was made. Histopathologically speaking, the term “pyogenic granuloma” is misleading because the illness is not linked to the production of granulomas. Pyogenic granuloma's etiopathogenesis is still unknown; true hemangioma is thought to be a reactive hyperproliferative of the vasculature brought on by a variety of stimuli; pyogenic granuloma may be caused by uneven angiogenic factor production in response to minor local trauma or cutaneous disease. Histopathological analysis and surgical excision are the methods used for diagnosis and treatment. The mainstay of treatment for granuloma pyogenic granuloma includes careful evaluation of any penile growth, thorough excision of the polypoid, histological examination, close follow‐up to check for relapse, and early management.

## Graphical abstract

Penile granuloma pyogenicum. Exhaustive examiantion of any penile growth with a focus on identifying external and local sources of irritants togeter with histological evaluation are the main stay of treatment of pyogenic granuloma.

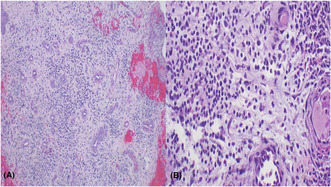

## 1 INTRODUCTION

Pyogenic granuloma is an acquired noncancerous vascular proliferation that arises from the mucosa and skin, seldom subcutaneously or intravascularly. It is also referred to as telangiectasis granuloma or lobular capillary hemangioma. It appears as a painless papule or lump that may bleed with or without minor trauma.^1^, ^2^ It usually takes the form of a polypoid and can be further classified into subtypes, including eruptive, dermal, intravascular, and subcutaneous. Head skin, oral mucosa, gingiva, trunk, neck, lower and upper extremities, and perianal are the most common places, with the genital areas being the least common.^3^ Vascular malformation, oral contraceptives, hormonal factors, pregnancy, and skin irritations resulting from trauma, poor hygiene, vasculitis, foreign objects, inflammatory skin conditions, oral retinoid therapy, and the antiretroviral medication indinavir are among the factors that predispose an individual to this condition. Recent research indicates that 7% of instances include mild trauma.^4^, ^5^, ^6^, ^7^, ^8^ It is astounding that microdamage during sexual activity is prevalent while genital pyogenic granuloma is uncommon.^9^, ^10^ We present a case of a 24‐year‐old male who presented at our facility with distal penile ulcerated polypoidal nodules.

## 2 CASE HISTORY

**FIGURE 1:**
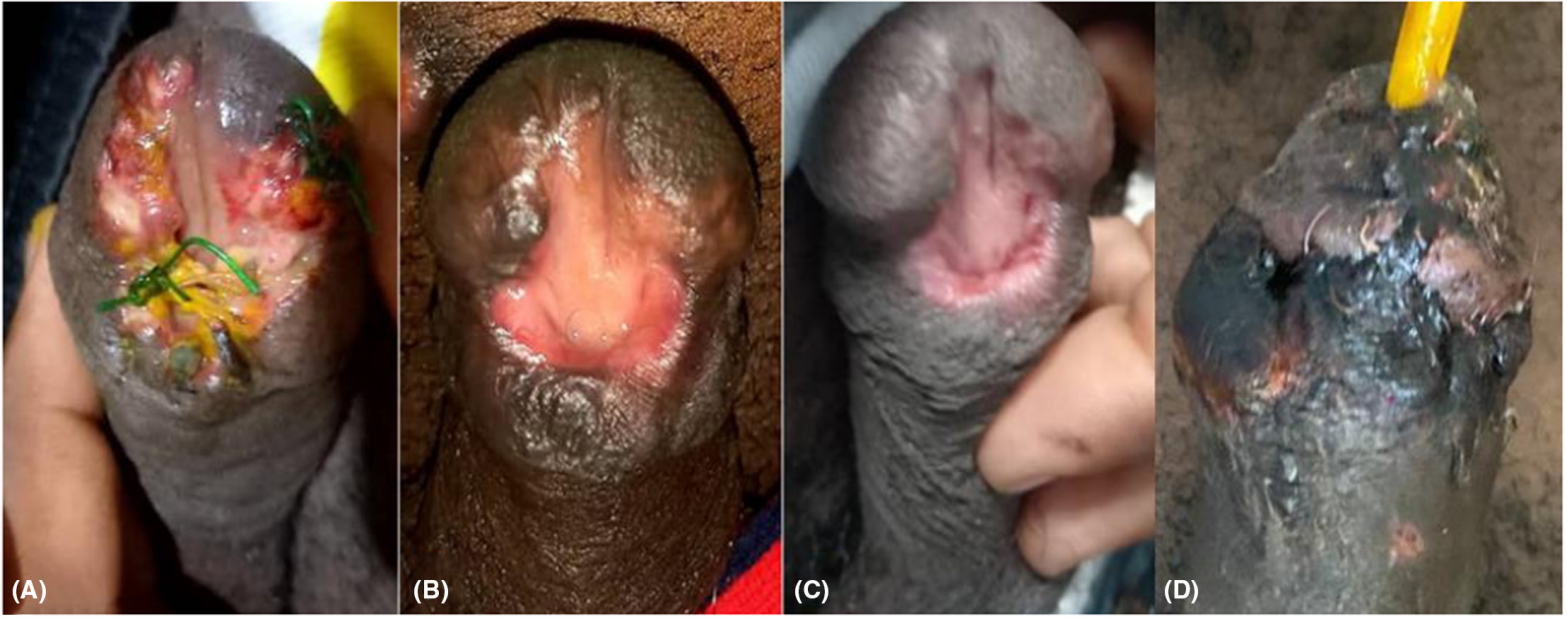
(A) Picture prior excision of the lesion showing eroded ventral part of the glans extending to sub coronal with some necrotic tissues, pus and sutures, exposing the navicularis fossa. (B) Two months post excision with completely healed wound. (C) 6 months postsurgical excision. (D) Post meatoplasty and glanuloplasty.

We discuss the case of a 24‐year‐old patient who had a ventral distal and glanular penile ulcerative lesion for 6 months and was seen at peripheral clinics. The lesion began as a small lump near the coronal and got worse over time, to the point where it became difficult for the patient to void. However, the patient denied any history of syphilis or relating the insult to sexual intercourse; a venereal disease research laboratory test was negative. The lump was ruptured at the health center, and two unsuccessful attempts to close the wound left the lesion extending to the glans and distal penile. Upon admission, a lobular pattern of vascular proliferation with inflammation and edema resembling granulation tissue was observed along the borders with limited pus discharge, the external urethral meatus at the subcoronal location, and suture materials from the peripheral hospital (Figure 1A).

## 3 METHODS

**FIGURE 2:**
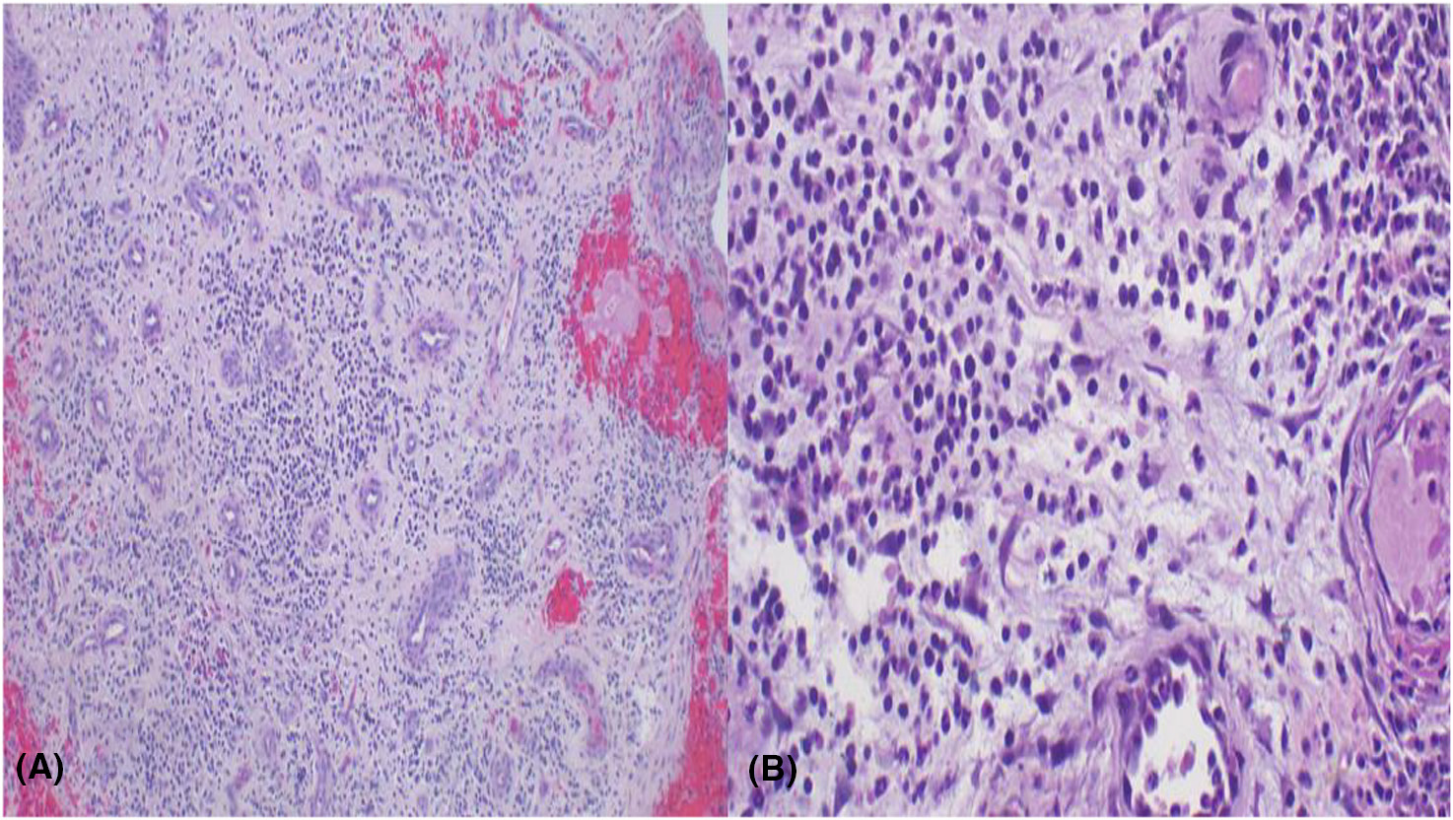
(A) Hisitopathology of pyogenic granuloma demonstrating a lobular pattern of vascular proliferation with inflammation and edema resembling granulation tissue, H&E staining 200× original magnification. (B) Photomicroscopy of pyogenic granuloma lesion demonstrating dense plasma cell inflammatory infiltrates; H&E staining 400× original magnification.

The patient was kept on antibiotics, excision of the polypoid margins of the wound was done, skin to mucosal approximation was not done, the edges of the urethral plate were allowed to granulate, and a biopsy was taken for histopathological study, which demonstrated dense plasma cell infiltrates which and lobular pattern of vascular proliferation with inflammation and edema resembling granulation tissue of the penile granuloma pyogenicum (Figure 2A,B). When this patient returned after 2 and 6 months, the penile wound had completely healed, as shown in (Figure 1B,C), respectively. However, the patient complained of an annoyingly spreading pee stream; therefore, we advanced the meatus near the tip of the glans of the penis, which was followed by the creation of glanular wings to allow glanuloplasty. (Figure 1D).

## 4 CONCLUSION

Comprehensive examination of any penile growth with a focus on identifying external and local sources of long‐term irritants, such as smegma, phimosis, and traumatizing events, avoiding squeezing or rupturing the polypoid lesion, and totally excising the growth, together with histological evaluation coupled with regular close follow‐up to detect early recurrence, are the mainstay of treatment for pyogenic granuloma. For our case urethral catheter was removed after 2 weeks post meatal advancement, 3 months later the patient was voiding well with single straight urine stream.

## 5 DISCUSSION

Histopathologically, the acronym “pyogenic granuloma” can be misleading because the condition itself is not linked to the development of granulomas.^3^ Inakanti Y et al provided a brief overview of the illness's history, beginning in 1844 with Hullihen's report on the first instance of pyogenic granuloma. In 1897, pyogenic granuloma was given the name Botryomycosis hominis; this is attributed to Poncet and Dor, and Hartzel is credited with coining the term “pyogenic granuloma” in the early 1900s.^11^

Pyogenic granuloma's etiopathogenesis is still undetermined; true hemangioma is thought to be reactive hyperproliferative of the vasculature brought on by a variety of stimuli; pyogenic granuloma's pathogenesis may involve uneven angiogenic factor production after mild local trauma or cutaneous disease.^12^ Biopsies are required for penile lesions that grow quickly due to unknown stimuli that cause endothelial proliferation and angiogenesis, present with unusual morphologies, and do not respond to anti‐wart treatment.^5^, ^13^ The table below shows several cases of penile pyogenic granuloma, depicting the location of the lesion in the penis, histology, and treatment given. Clinical differential diagnoses of pyogenic granuloma are pyoderma gangranosum of the penis, urethral caruncle, urethral prolapse, angiokeratoma, genital warts, and cherry angioma.^11^

Treatment options for pedicunculated pyogenic granuloma comprise cryotherapy, cauterization and curettage, or diathermy coagulation of the base. While pulsed dye lasers can be used to manage small lesions, electrodesication is not always the best option as it can cause irreversible changes. Squeezing the lesion is discouraged, and when recurrence occurs, the best course of action is to excise a thin, deep ellipse underneath the lesion; imiquimod and timolol are suggested as effective topical therapies.^5^, ^6^, ^8^, ^10^ There is a possibility for recurrence irrespective of the method utilized to remove the lesion, particularly if it was not entirely excised or if there are recurring irritations such as phimosis, smegma, or slight trauma.^14^

There aren't many cases of penile pyogenic granuloma in the literature, and most of them involve children and young adults. Histopathological evaluation and surgical excision are used to diagnose it; prior to excision, an ultrasound scan could show a vasculogenic lesion.^7^, ^9^ A biopsy must be collected for a histopathological study in order for an expert to properly diagnose this dermatological disorder (Table 1).^14^

**TABLE 1:** Showing location of the lesion in the penis, histology, and treatment given.

Reference	Age(year)	Treatment	Follow‐up (month)	Histopathology
Yumnam et al^8^	8	Surgical excision	6	Proliferating vessels surrounded by a mixed inflammatory infiltrate comprising lymphocytes, plasma cells, neutrophils, and eosinophils.
Sheetansu K et al^2^	Middle aged	Surgical excision	–	Lobular proliferation of capillaries with thinned out epithelium.
Akan S^7^	69	Surgical excision	24	Ulcerated surfaced squamous epithelium Lobular vascular capillary structures beneath squamous epithelium
Katmeh FR et al^9^	37	Surgical excision	3	Encapsulated, relatively well defined dermal based lobular proliferation of small, thick‐walled vessels extending to the upper part of the corpus cavernosum, surrounded by mixed inflammatory infiltrate.
Claudio S et al^1^	4	Surgical excision	6	Edematous stroma with a modest chronic inflammatory infiltration imbedded in congested capillaries and venules comprised the angiomatous tissue.
Our case	24	Surgical excision	6	Granulation tissue‐like edema and inflammation accompanying a lobular pattern of vascular proliferation

The aforementioned table lists examples of pyogenic granuloma cases that were surgically treated and followed for an average of 6 months. All of these cases did not have a recurrence and had common histological characteristics, including a lobular pattern of vascular proliferation and inflammatory infiltration.

## AUTHOR CONTRIBUTIONS

**Gideon Safiel Mwasakyalo:** Conceptualization; methodology; supervision; writing – original draft; writing – review and editing. **Frank Bright:** Writing – review and editing. **Orgenes Jasper Mbwambo:** Writing – review and editing. **Bartholomeo Nicholaus Ngowi:** Writing – review and editing. **Daniel Mwakibibi:** Writing – review and editing. **Jasper Saidi Mbwambo:** Writing – review and editing. **Musa Raymond Majura:** Writing – review and editing. **Evans Azina Sanga:** Writing – review and editing. **Alex Mremi:** Methodology; writing – review and editing.

## FUNDING INFORMATION

We now disclose that we have received no funding for this work.

## CONFLICT OF INTEREST STATEMENT

The writers have no competing interests.

## CONSENT

Written informed consent was obtained from the patient to publish this report in accordance with the journal's patient consent policy.

## Data Availability

There was no generated data in this study.
